# Lessons Learned from Quantitative Dynamical Modeling in Systems Biology

**DOI:** 10.1371/journal.pone.0074335

**Published:** 2013-09-30

**Authors:** Andreas Raue, Marcel Schilling, Julie Bachmann, Andrew Matteson, Max Schelke, Daniel Kaschek, Sabine Hug, Clemens Kreutz, Brian D. Harms, Fabian J. Theis, Ursula Klingmüller, Jens Timmer

**Affiliations:** 1 Institute of Physics, University of Freiburg, Freiburg, Germany; 2 Institute of Computational Biology, Helmholtz Center, Munich, Germany; 3 Systems Biology of Signal Transduction, German Cancer Research Center-Center for Molecular Biology Alliance, Heidelberg, Germany; 4 Department of Computational Biology, New York University, New York, New York, United States of America; 5 Center for Mathematical Sciences, Technical University Munich, Garching, Germany; 6 Merrimack Pharmaceuticals, Cambridge, Massachusetts, United States of America; 7 Freiburg Institute for Advanced Studies, University of Freiburg, Freiburg, Germany; 8 Zentrum für Biosystemanalyse, University of Freiburg, Freiburg, Germany; 9 BIOSS Centre for Biological Signalling Studies, University of Freiburg, Freiburg, Germany; National Institute of Genomic Medicine, Mexico

## Abstract

Due to the high complexity of biological data it is difficult to disentangle cellular processes relying only on intuitive interpretation of measurements. A Systems Biology approach that combines quantitative experimental data with dynamic mathematical modeling promises to yield deeper insights into these processes. Nevertheless, with growing complexity and increasing amount of quantitative experimental data, building realistic and reliable mathematical models can become a challenging task: the quality of experimental data has to be assessed objectively, unknown model parameters need to be estimated from the experimental data, and numerical calculations need to be precise and efficient.

Here, we discuss, compare and characterize the performance of computational methods throughout the process of quantitative dynamic modeling using two previously established examples, for which quantitative, dose- and time-resolved experimental data are available. In particular, we present an approach that allows to determine the quality of experimental data in an efficient, objective and automated manner. Using this approach data generated by different measurement techniques and even in single replicates can be reliably used for mathematical modeling. For the estimation of unknown model parameters, the performance of different optimization algorithms was compared systematically. Our results show that deterministic derivative-based optimization employing the sensitivity equations in combination with a multi-start strategy based on latin hypercube sampling outperforms the other methods by orders of magnitude in accuracy and speed. Finally, we investigated transformations that yield a more efficient parameterization of the model and therefore lead to a further enhancement in optimization performance. We provide a freely available open source software package that implements the algorithms and examples compared here.

## Introduction

Biological processes such as the regulation of cellular decisions by signal transduction pathways and subsequent target gene expression are governed by highly complex molecular mechanisms. These intertwined processes are difficult to understand by interpreting experimental results directly since the underlying mechanism can be rather counter-intuitive. In the context of Systems Biology, dynamical models consisting of ordinary differential equations (ODE) are a frequently used approach that facilitates to analyze the mechanism of action in a systematic manner. For example, the cellular response to perturbations in the molecular reactions can be investigated. The advantage of building a mathematical model is that molecular mechanisms that are supposed to govern the respective process need to be formulated explicitly. This allows to test hypothesis about the supposed network structure of the molecular interactions [1] and to predict systems behavior that is not accessible by experiments directly [2]. However, the bottle neck for successful mathematical description of cell biological processes are efficient and reliable numerical methods. In the following we introduce quantitative dynamical modeling and subsequently present results on how challenges in the model building and calibration process were tackled.

### Modeling the dynamics of cellular processes

The majority of cellular processes can be described by networks of biochemical reactions. The dynamics of these processes, i.e. the time evolution of the concentrations of the involved molecular compounds, can often be modeled by systems of ODEs [3]

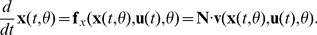
(1)


The variables 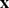 correspond to the dynamics of the concentration of 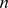 molecular compounds such as hormones, proteins in different phosphorylation states, mRNA or complexes of the former. The right hand side of Equation (1) can usually be decomposed into a stoichiometry matrix 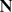 and reaction rate equations 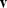 of the molecular interactions [4]. A time dependent experimental treatment that alters the dynamical behavior of the system can be incorporated by the function 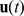. For example, this can be the extracellular concentration of a hormone that is degraded during the experiment or is manually controlled by the experimenter over time. The initial state of the system is described by 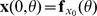. Often, these initial conditions represent a steady state solution to Equation (1) that indicates that the system is in equilibrium in the beginning of the experiment. The set of parameters 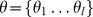 contains reaction rate constants and initial concentrations of the molecular compounds that fully determine the simulated dynamics.

ODE models assume spatial homogeneity inside the compartments of the cell, i.e. that diffusion and active transport are fast compared to the reaction rates of molecular interactions and the spatial extent of the compartment. Furthermore, such models describe macroscopic dynamics. Intrinsic stochasticity caused by the discrete nature of the reactions is usually not considered. Extrinsic stochasticity [5] caused by cell to cell variability can be considered if single cell data is available. If necessary, the class of ODE models can be extended to consider both sources of stochasticity [6].

### Biological context of modeling examples

**Figure 1:**
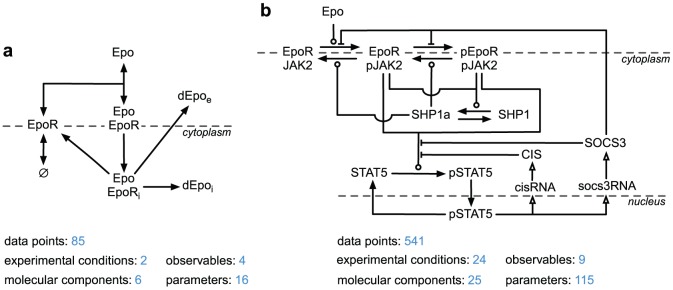
Quantitative dynamic models describing erythropoetin signaling used as examples. The hormone erythropoietin (Epo) is the key regulator of erythropoiesis, the production of red blood cells. (**a**) Epo receptor model [2]. The model describes the interaction and the trafficking of the hormone and of its membrane receptor (EpoR). The active complex Epo_EpoR can be internalized (Epo_EpoR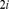) and is either recycled back to the cell membrane or is degraded (dEpo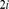, dEpo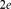). (**b**) Model of Epo induced JAK2/STAT5 signaling [7]. In erythroid progenitor cells (CFU-E), the hormone Epo induces activation of the tyrosine kinase Janus kinase 2 (JAK2). Subsequently, the signal transducer and activator of transcription 5 protein (STAT5) is activated and shuttles to the nucleus where it induces target gene expression. Two of the target genes encode for the negative feedback regulators suppressor of cytokine signaling 3 (SOCS3) and cytokine-inducible SH2-containing protein (CIS).

**Figure 2:**
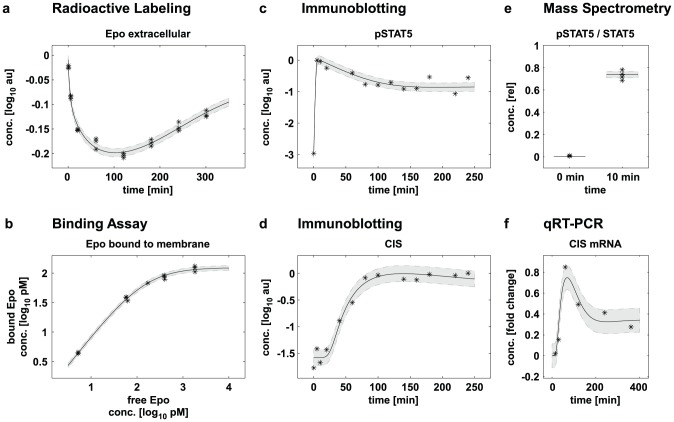
Data obtained by different experimental approaches, associated measurement noise and corresponding model simulation of the pathway dynamics. Solid lines indicate the simulated model dynamics with the optimal parameter values. Gray shading indicates one standard deviation of the measurement noise that is associated with the respective measurement technique. (a,b) Data for Epo receptor model [2], 36 out of 85 total data points used for parameter estimation are displayed. (c,d,e,f) Data for JAK2/STAT5 model [7], 92 out of 541 total data points used for parameter estimation are displayed.

Two recent applications, differing in the size of available experimental data sets and of the mathematical model, were used as examples (Figure 1). The first example is a mathematical model describing the binding of the hormone erythropoetin (Epo) to its membrane receptor (EpoR) and its subsequent trafficking [2]. The second example is a model of the Epo-induced JAK2/STAT5 signaling pathway that primarily consists of the cytoplasmic tyrosine kinase JAK2 and the latent transcription factor STAT5 [7]. The investigated processes, which range from interactions of the ligand with the receptor to transcriptional induction of negative feedback regulators, were addressed by different experimental approaches. Selected examples of the experimental data and of the model simulations are displayed in Figure 2. For the Epo receptor model, radioactively labeled ligand was used to monitor Epo concentrations in different compartments of the cell. Binding assays were employed to infer receptor binding affinities of Epo. For the JAK2/STAT5 model, data was generated (i) by quantitative immunoblotting, yielding time-course data for proteins and the respective phosphorylations [8], (ii) by qRT-PCR providing time resolved measurements on mRNA expression and (iii) by quantitative mass spectrometry that revealed relative phosphorylation degrees [9]. Absolute protein concentrations were determined by employing serial dilutions of protein standards in the immunoblotting approach. Furthermore, different experimental conditions, such as increasing ligand concentrations and treatment with inhibitors, were used. With these qualitatively and quantitatively different data the mathematical models could be established as well as calibrated more reliably.

### Quantitative measurements for model calibration

Often, not all desired molecular compounds can be measured directly or individually. Immunoassays are limited by the availability and specificity of antibodies. For example, phosphorylation of single proteins and proteins bound in complexes can often not be distinguished by immunoblotting measurements, only their sum may be available. In order to compare the model dynamics simulated for candidate parameter values 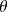, see Equation (1), to these experimental data, the dynamic variables 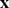 are mapped to 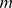 observables
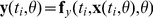
(2)via a function 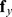. The observables 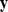 are the quantities that can be measured in experiments at time points 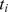. They may depend on additional parameters that are included in 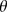 such as scaling or offset parameters in case of relative data or measurement background. Typically, the number of observables 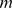 is smaller than the number of molecular compounds 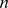.

For each observable 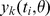 the corresponding experimental data 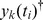 contains measurement noise 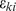. For concentration measurements by biochemical assays it is reasonable to assume that the measurement noise is multiplicative log-normally distributed. For data generated by immunoblotting experiments it was explicitly shown that the measurement noise is log-normally distributed [10]. In addition, biological variability that is contained in the data was shown to be log-normally distributed as well [11]. For parameter estimation additive normally distributed measurement noise is more convenient. A log-transformation of both experimental data and observables yields additive normally distributed measurement noise 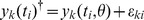 with 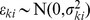. In the following, 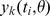 and 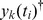 already denote log-10 transformed values. However, the variance components 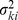 of the measurement noise are often not known *a priori*.

### Model calibration by maximum likelihood estimation

In order to calibrate the dynamical model, the observables 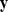, see Equation (2), are compared to experimental data 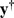. For normally distributed measurement noise the likelihood
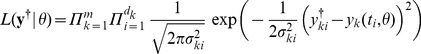
(3)of the experimental data given the model parameters 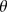 is a well-known distance measure [12]. Here, 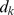 denotes the number of experimental data 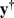 for each observable 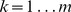, measured at time points 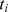 with 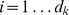. 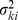 are the variance components of the measurement noise of each data point. By maximizing 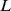 the maximum likelihood estimates of the unknown parameters 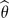 can be obtained [13]. It is more common, equivalent and numerically more efficient to minimize the negative logarithm of the likelihood function 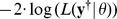 instead. For parameters that are by definition non-negative a log-scale should be used in the parameter estimation. This facilitates parameters being potentially different by orders of magnitude to be handled with equal efficiency by numerical computations. This applies for most of the parameters occurring in ODE models such as reaction rate constants, initial concentrations, scaling and offset parameters. The allowed search space for most of the parameters spanned six orders of magnitude and was set to 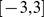 on a log10-scale. Due to the non-linearity of the optimization problem numerical algorithms have to be used to estimate the parameters.

## Results

### Efficient numerical simulation of quantitative dynamic models

The system of nonlinear ODEs, see Equation (1), that implements the biological processes has to be solved numerically. The time scales for the reaction rate of the molecular processes can differ by orders of magnitude. Therefore, a stiff ODE solver should be applied. We used the CVODES algorithm [14] that was coded in C for efficiency in combination with a MATLAB mex-interface. For each of the experimental conditions the ODE system has to be modified according to the applied treatment. For instance for the JAK2/STAT5 model, an additional treatment with Actinomycin D inhibits transcription of negative feedback regulators SOCS3 and CIS and therefore modifies the ODE system. Consequently, there can be as many variants of the ODE systems as experimental conditions, each having a different numerical solution for the dynamics. For one comparison of the whole model to the experimental data, given a specific set of candidate parameters, all ODE variants have to be solved. During parameter estimation many evaluations of the whole model are necessary. This can be a numerically intensive task. All ODE variants can be solved independently, therefore this problem is ideal for parallel computing. We employed a multithreading technique that results in a significant acceleration on multi-core machines.

**Figure 3:**
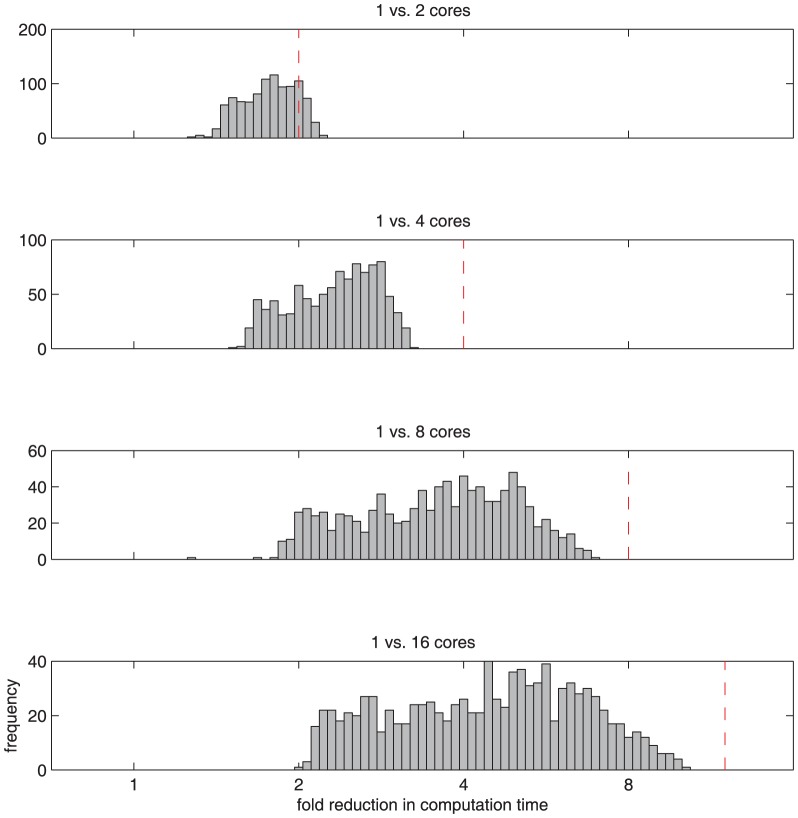
Acceleration of numerical computations for the solution of ODE systems by multithreading. For each bar in the figure the 24 variants of the ODE systems used for the JAK2/STAT5 model were solved for 1000 randomly drawn sets of parameters using a Latin hypercube sampling strategy. The theoretically possible acceleration is displayed by the red dashed line.

For the JAK2/STAT5 model, 24 ODE variants have to be solved concurrently. In this case, a 2-core machine yields an acceleration of approximately 2-fold, a 4-core machine yields an acceleration of approximately 2 to 3-fold, an 8-core machine yields an acceleration of approximately 2 to 6-fold (Figure 3). For more cores, the overhead due to thread creation and different runtimes of single threads limit the acceleration of the calculations.

### Importance of the assessment of measurement noise

In the quantitative dynamic modeling approach, the mathematical model is calibrated based on experimental data. Experimental data contain measurement noise. For a realistic calibration of the dynamical model an adequate assessment of the measurement noise is important. The magnitude of the noise depends on the measurement technique (Figure 2). For instance, antibodies used in immunoblotting differ with respect to their specificity. Furthermore, the noise level is influenced by the biological context of the experiment. For example, in experiments with primary cells, available material is limited compared to experiments using cell lines. In addition, biochemical assays such as immunoblotting, qRT-PCR or mass spectrometry are time and cost intensive. Frequently, no more than three repetitions of an experiment are available, which results in a challenging setting for reliable parameter estimation.

The variance 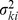 of the measurement noise controls the parameter estimates 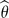 by entering into the likelihood, see Equation (3). Therefore, a realistic choice of the variance components 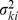 is crucial in order to obtain reliable estimates of the model parameters and of the uncertainties that are associated with this estimation process. Two approaches for the assessment of measurement noise were investigated:

1. A standard approach is the estimation of variance directly from measurement replicates as a *pre-processing* of the data. However, in the case of few measurement replicates this approach leads to highly variable estimates. In the case of single replicates it is not feasible.
2. Alternatively, the variance of the measurement noise can be estimated *simultaneously* with the model dynamics. To this end the distribution of the measurement noise is considered as a parametrized function
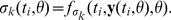
(4)

These additional parameters that represent the magnitude of the noise are estimated simultaneously with the remaining model parameters. This approach facilitates an objective and automated estimation of the measurement noise and of the actual model parameters. Reasonable results can also be obtained if no measurement repetitions are available. The measurement noise estimated by this approach is depicted in Figure 2 by gray shading.

**Figure 4:**
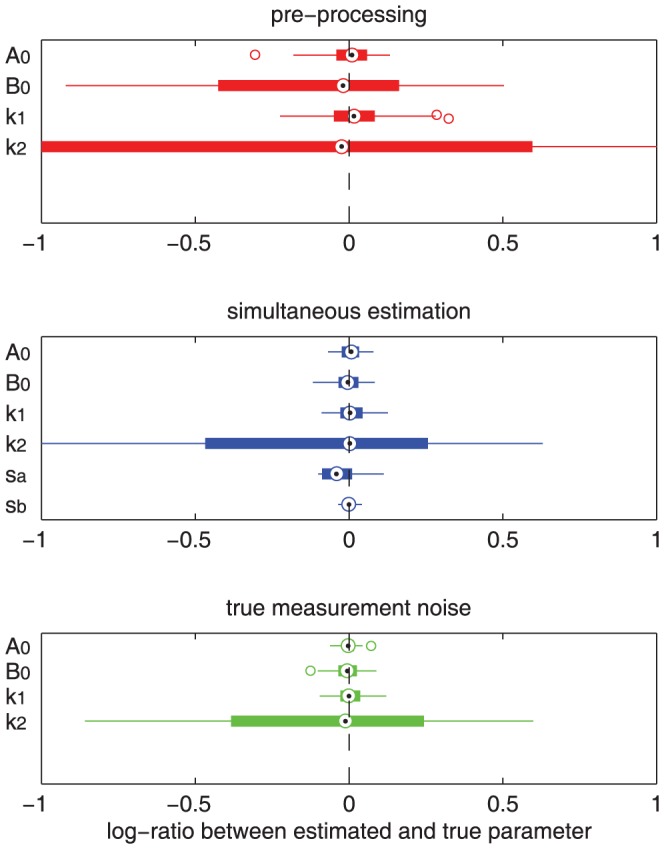
Analysis of the bias and variance of parameter estimates induced by different methods for the assessment of measurement noise. The figure shows the result of two hundred independent parameter estimation runs for one hundred different sets of simulated data for the parameters A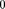, B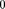, k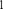, k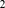 and 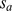, 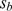 where applicable. The amount of measurement noise was assessed by a pre-processing approach (a), by using a simultaneous estimation of dynamics and measurement noise (b) and using the true values for 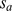 and 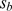 in the estimation (c).

To investigate the performance of both approaches systematically, i.e. analysis of bias and variance induced to parameter estimation, a simulation study where true parameter values are known is used. A simple test case with two reactions, A 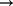 B with rate constant k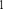 and B 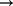 A with rate constant k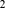 is investigated. Also initial conditions A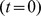  = A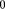 and B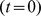  = B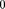 are estimated from the data. Experimental data is simulated for 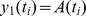 and 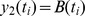 for time points 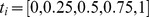 in triplicates and with absolute and relative measurement noise according to 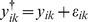 where 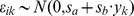, 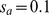 and 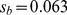. For the pre-processing approach, for each time point 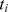 and 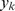 the variance 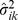 is individually estimated from the triplicates and is used in the parameter estimation by 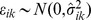. For the simultaneous estimation, the parametrized function 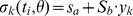 and 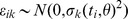 is used in the parameter estimation. Here, 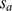 and 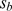 are included in 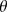 and are estimated simultaneously with the remaining parameters. Both approaches yield unbiased results, however, the pre-processing approach induces a considerably higher amount of variance in the estimated parameters than the simultaneous estimation (Figure 4a,b). Even estimation of the parameters using the true values for 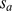 and 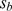 is not significantly better than the simultaneous estimation approach (Figure 4c).

The results show that the simultaneous calibration of model dynamics and measurement noise in a single step facilitates a statistically more accurate assessment of the model parameters than using a preprocessing of the experimental data.

### Performance of numerical optimization algorithms

To calibrate a model, all unknown parameters, including the parameters contained in the measurement noise distribution, have to be estimated based on the experimental data. Parameter estimation involves an optimization routine that varies all unknown parameters to obtain the best possible representation of the data by the model. The optimal parameter values can be ascertained by maximum likelihood estimation. Minimizing the negative logarithm of the likelihood is mathematically equivalent to maximizing the likelihood because the logarithm is a monotonically increasing function. Minimization of log-likelihood provides the same parameter estimates, but it has significant advantages in efficiency. In the following, we will refer to negative log-likelihood as objective function. Given two sets of parameters, the one that has the smaller associated objective function is the one that provides better agreement between a model and experimental data.

**Figure 5:**
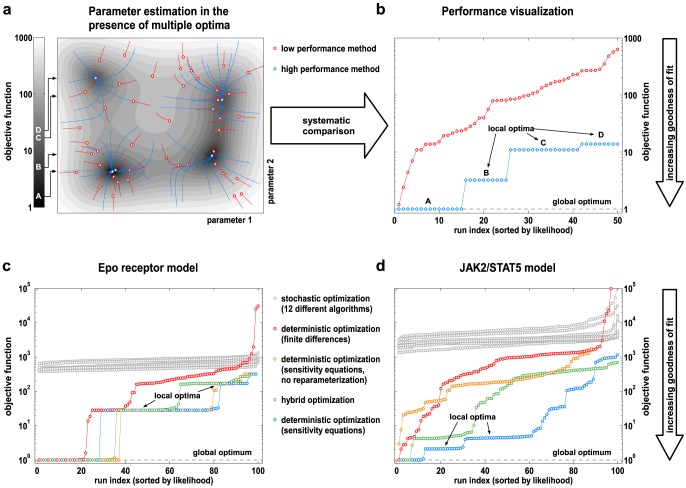
Performance analysis of parameter estimation using numerical optimization methods. (**a**) A two dimensional parameter estimation problem [15] bearing multiple optima (global: A; local: B,C,D) is displayed for illustrative purposes. Traces in parameter space of two hypothetical methods with high (blue) and low performance (red) are displayed. 50 independent runs with each method are displayed; the circles indicate the results of the estimation. (**b**) The visualization of optimization performance by sorting objective function values increasingly is also possible for high dimensional problems. It reveals that the performance of the red method is low, i.e. results are unreliable, whereas the performance of the blue method is high, i.e. results are reproducible and reliable. (**c,d**) Visualization of performance using 100 independent optimization runs with each of the considered algorithms for both quantitative dynamic models. For illustrative reasons, the global optimum was centered to one. For stochastic optimization (gray), 12 different algorithms [18] were used (see Figure 11 for details). For deterministic optimization, two different approaches for the calculation of derivatives were compared: (i) finite difference approximation (red) and (ii) analytically derived sensitivity equations (orange and blue). Initial guesses for the parameters were generated by Latin hypercube sampling [17]. All algorithms are described in Table 1.

A common problem in the parameter estimation of dynamic models is the occurrence of multiple optima, i.e. multiple basins of attraction in the objective function. As an illustrating example [15], an objective function of a two dimensional parameter space that contains four optima, consisting of one global optimum (A) and three local optima (B,C,D), is displayed in Figure 5a. In general, there are three approaches to this problem:

1. *Stochastic optimization algorithms* apply sophisticated heuristics that randomly sample parameter space to evaluate the objective function. Due to the stochastic approach these methods are less likely to get stuck in local minima. This is particularly advantageous for applications that are characterized by many local optima. Hence, they increase the probability of locating the global optimum despite local optima. Typically, these methods do not evaluate derivatives of the objective function.
2. *Deterministic optimization algorithms* take steps that successively decrease the value of the objective function beginning from an initial guess for the parameter values [16]. They evaluate derivatives of the objective function. This leads to more rapid convergence to the optimum compared to stochastic algorithms. However, depending on the initially assumed parameter values a deterministic optimization algorithm may converge to a local rather than global optimum. This limitation of deterministic optimization algorithms can be overcome by performing many independent optimization runs from randomly selected initial parameter guesses. This “multi-start” approach facilitates a broad coverage of the parameter search space in order to find the global optimum. Latin hypercube sampling [17] of the initial parameter guesses can be used to guarantee that each parameter estimation run starts in a different region in the high-dimensional parameter space. This method prohibits that randomly selected starting points are accidentally close to each other (Materials and Methods: Latin hypercube sampling).
3. *Hybrid optimization algorithms* use a combination of both strategies. First, promising candidate sets of parameter values are generated using a stochastic strategy. The candidate sets are then further improved by a deterministic strategy.

We have performed a comprehensive comparison of 15 optimization methods selected from the above categories (Table 1) using both examples. 12 different stochastic optimization algorithms [18] were investigated (Materials and Methods: Stochastoc optimization algorithms). For comparison of deterministic methods, we applied a trust region algorithm [19] in combination with Latin hypercube sampling and two different approaches for calculating derivatives of the objective function: *finite difference approximation* and analytically derived *sensitivity equations*. Additionally, we have evaluated the hybrid algorithm scatter search [20], [21]. All algorithms were applied with default settings that are recommended for generic applications.

**Table 1:** Description of optimization algorithms.

type	name	description
stochastic	STD-ES	standard evolutionary strategy
stochastic	CMA-ES	evolutionary strategy with covariance matrix adaption
stochastic	STD-GA	standard genetic algorithm
stochastic	PSO	particle swarm optimization with constriction
stochastic	DE	differential evolution algorithm
stochastic	TRIBES	adaptive particle swarm optimization
stochastic	RANDOM	random Monte-Carlo search
stochastic	HILLC	hill climbing strategy
stochastic	CBN-ES	cluster-based niching evolutionary strategy
stochastic	CHILL	clustering hill climbing strategy
stochastic	IPOP-CMA-ES	evolutionary strategy with increasing population size
stochastic	CBN-GA	cluster-based niching genetic algorithm
deterministic	LSQNONLIN FD	trust region algorithm using Latin hyper cube multi-starts
		and finite difference approximation for derivative calculations
deterministic	LSQNONLIN SE	trust region algorithm using Latin hyper cube multi-starts
		and sensitivity equations for derivative calculations
hybrid	SSmGO	enhanced scatter search using FMINCON
		for deterministic optimization

Stochastic optimization algorithms are provided by the *Evolutionary Algorithms Workbench* [18]. LSQNONLIN and FMINCON are part of the *Optimization Toolbox* (MATLAB, R2011a, The Mathworks Inc., Natick, MA). SSmGO is part of the toolbox *Scatter Search for Global Optimization for Matlab* [20], [21].

To evaluate their reliability, each method was applied 100 times with different initial parameter guesses. Ideally, each optimization run should reach an optimum, either local or global. Reliable methods are characterized by returning the same results reproducibly, for instance the global optimum A and the local optima B,C and D (Figure 5a,b). This performance evaluation enables characterization of multiple optima and visualization of results for high dimensional problems. The number of repetitions of optimization should be chosen large enough, until the best method yield reproducible results in a satisfactory number of cases. In our examples 100 repetitions were large enough. By this method, multiple optima can clearly and reproducibly be distinguished for both examples. In the Epo receptor model, we discovered three local optima. In the JAK2/STAT5 model, five local optima were detected. For the smaller Epo receptor model, the hybrid optimization algorithm performed equally well, but the method had a considerably higher computational cost. For multi-start deterministic optimization, the rate of convergence to the global optimum is associated with the size of the basin of attraction of the global optimum compared to the size of the entire parameter search space. For the Epo receptor model, the fraction is about 30–40%, for the JAK2/STAT5 model only about 10%. Our comparison indicates that multi-start deterministic optimization using the sensitivity equations for derivative calculations performs best for both examples (Figure 5c,d) and is appropriate for these examples.

**Figure 6:**
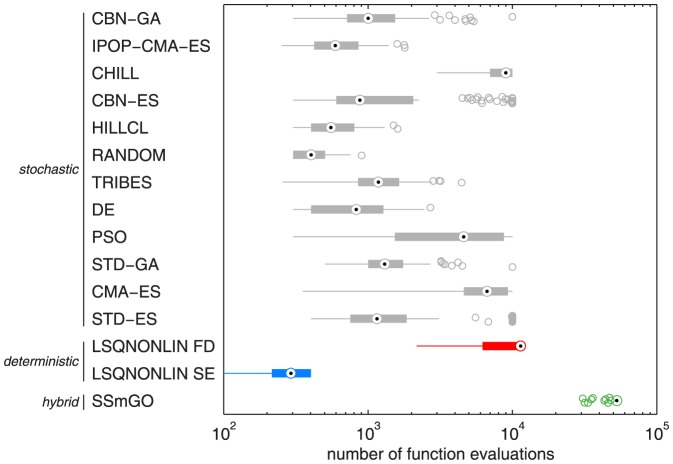
Number of calls to the objective function for 100 independent optimization runs with each of the considered algorithms for the JAK2/STAT5 model. For description of the algorithms, stochastic optimization (gray), deterministic optimization (red, blue) and hybrid optimization (green) see Table 1. The LSQNONLIN algorithm (MATLAB, R2011a, The Mathworks Inc., Natick, MA) was used in combination with Latin hyper cube sampling of the initial parameter guesses and using two different approaches for derivative calculation, finite difference approximation (FD) and the sensitivity equations (SE). LSQNONLIN using SE is the most efficient algorithm for parameter estimation considered in this study. For all algorithms default options were used. The maximum number of allowed function evaluations 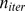 was chosen such that the algorithm stops before reaching 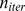 in most cases. For stochastic optimization (gray) 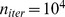, for deterministic optimization using SE (red) 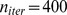 and using FD (blue) 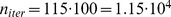 and for hybrid optimization (green) 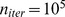.

All optimization algorithms were also compared in terms of computational speed. This showed that multi-start deterministic optimization using the sensitivity equations is also the computationally most efficient approach (Figure 6).

### Derivatives used in deterministic optimization algorithms

**Figure 7:**
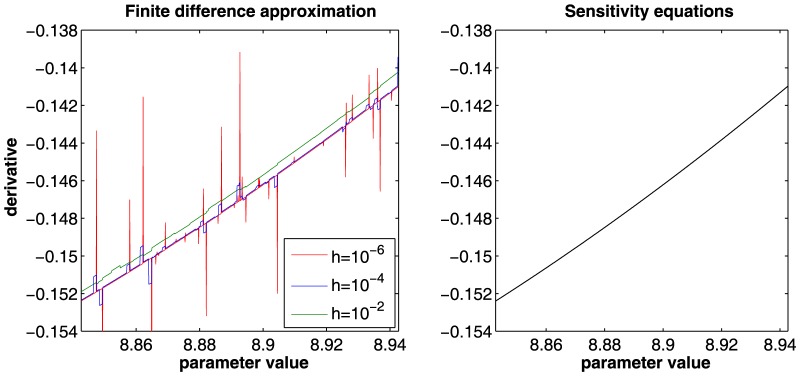
Comparison of derivatives calculated by finite difference approximation and by the sensitivity equations. The figure shows the calculation of derivatives 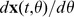 for the JAK2/STAT5 model for the dynamical variable pSTAT5 at 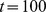 minutes with respect to the parameter CISInh on the y-axis, for several values of the parameter CISInh on the x-axis. For finite difference approximation, 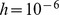 (red), 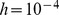 (blue) and 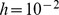 (green) was used. The numerical errors rapidly become uncontrollable as 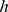 gets smaller. For larger 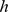, finite difference approximation is of bad quality showing systematic errors. In contrast, the analytical derivatives calculated by the sensitivity equations produce stable and correct results (black).

Deterministic optimization algorithms such as LSQNONLIN (MATLAB, R2011a, The Mathworks Inc., Natick, MA) that is applied here require derivatives of the objective function, see Equation (3), with respect to parameters. It is important to use reliable and efficient numerics for the calculation of the derivatives because they will guide the optimization method to the optimum. The inner derivatives 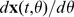, also called *sensitivities*, are required for this calculation. Two approaches for the calculation of the sensitivities were investigated:

1. *Finite difference approximation* is a standard approach where the model trajectories 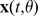 are calculated for perturbed parameters
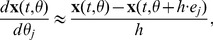
(5)here, 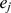 is the 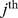 unit vector and 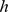 should be chosen sufficiently small. For ODE models this approach leads to significant numerical instabilities because 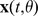 can only be approximated numerically by the ODE solver. An absolute and relative tolerance of 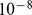 was used here. As 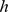 should be small, the numerical error in the difference of two noisy solutions 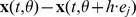 can dominate (Figure 7). Note that there is no generic way of choosing 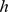 that leads to least errors.
2. *Sensitivity equations* represent additional ODEs
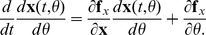
(6)for the derivatives [22] that are solved simultaneously with the original ODE system, see Equation (1). An efficient algorithm for solving the enlarged ODE system is the CVODES solver [14]. There are some numerical properties that enable to increase the performance of solving the enlarged ODE system. For example, the sensitivity equations inherit the stiffness of the original ODE system, therefore the adaptive step size control for the enlarged ODE system can be chosen equal to that of the original system. Futhermore, the Jacobian matrix of the right hand side of the sensitivity equations is composed block by block out of the Jacobian matrix of the original ODE system. In our simulations calculating derivatives by sensitivity equations is ten times faster than by finite difference approximation.

Our results show that using finite difference approximation of the derivatives in the optimization leads to unreliable results (Figure 5c,d). Numerical inaccuracies in the derivatives (Figure 7) are responsible for inaccuracy in the search direction of the algorithm and consequently lead to premature termination of the optimization procedure [23]. The calculation of derivatives using the sensitivity equations is not only more reliable but also considerably faster. For the JAK2/STAT5 model the calculation is approximately ten times faster compared to finite difference approximation.

### Decoupling of parameters using scaling invariances

Each ODE model that corresponds to a reaction network is realized as equations that describe quantities carrying physical units. Such an ODE model has an intrinsic scaling invariance originating from the free choice of units. This invariance can be exploited to disentangle parameters that carry time units such as rate constants and concentration units such as initial concentrations, dissociation constants or scaling parameters. The corresponding reparameterization does not restrict the dynamics of the model and is also known as nondimensionalization [24].

**Figure 8:**
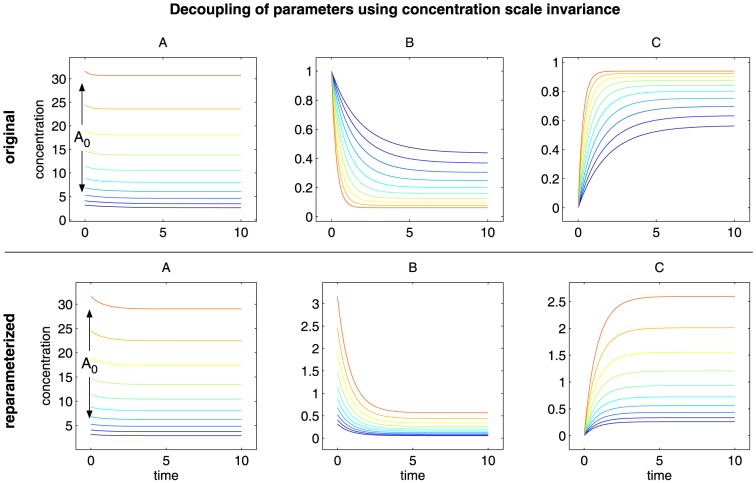
Decoupling of parameters using concentration scale invariance. The figure shows the dependency of the dynamics of the original system and of the reparameterized system on the parameter A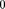. In case of the original system (top row) the parameter controls the scale of species A but also the time when the steady state is reached. In case of the reparameterized system (bottom row) the parameter controls only and exclusively the concentration scale of the entire system leaving the shape of the dynamics unaffected.

**Figure 9:**
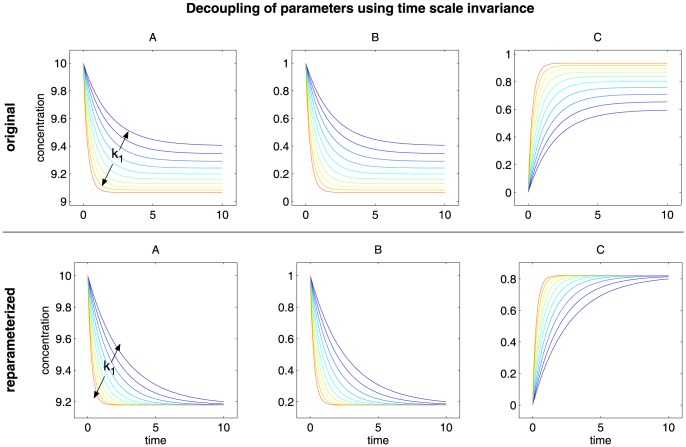
Decoupling of parameters using time scale invariance. The figure shows the dependency of the dynamics of the original system and of the reparameterized system on the parameter k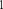 and k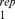 respectively. In case of the original system (top row) the parameter controls the level of the steady state but also the time when the steady state is reached. In case of the reparameterized system (bottom row) the parameter controls only and exclusively the time scale of the entire system leaving the shape of the dynamics, especially the steady state level, unaffected.

Lets consider a simple test case with two reactions, A+B 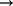 C with rate constant k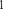 and C 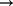 A+B with rate constant k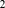 for illustrative purpose. The initial conditions A 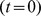  = A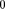 and B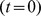  = B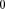 are considered as free parameter whereas it is assumed that C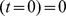. The reparameterizations that implement the invariance can be derived by dimensional analysis of the parameters. The parameter 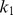 is expressed as concentration/time, 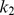 as 1/time, while A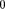 and B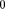 are expressed as concentration. Without loss of generality, we pick A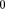 to represent the concentration scale and use reparameterizations B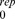  =  B 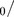 A 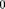 and k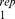  = k
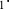
A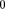. The new parameters B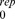 and k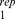 do not carry the concentration units any more, B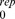 is dimensionless and the unit of k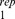 is 1/time. Similarly and without loss of generality, we pick k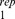 to represent the time scale and use the reparameterization k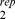  = k
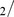
k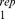 where the new parameter k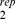 is now dimensionless. As results of the reparameterization, the concentration and time scales of the dynamics are disentangled
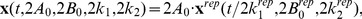
(7)i.e. the concentration scale is exclusively controlled by A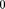 and the time scale is exclusively controlled by k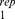 (Figure 8 and 9). The parameter transformations as well as the ordinary differential equations for the Epo receptor model and for the JAK2/STAT5 model are given in the Materials and Methods.

For most of the measurement techniques, the observables 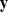 are related to the dynamical variables 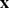 by scaling factors 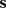


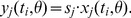
(8)


Often, only relative data can be obtained, e.g. of mRNA concentration by qRT-PCR or of protein concentration by immunoblotting. In this case, the respective parameter 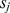 is unknown and cannot be determined from this experimental data. The concentration scale invariance illustrated in Equation (7) and Equation (8) directly imply that also the product 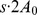 cannot be determined. Both 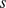 and 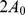 are structurally non-identifiable [25] in this case. This structural non-identifiability can directly be detected from the reparameterization. As a consequence, model predictions for quantities 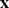 that lack experimental information about the absolute concentration can only be made on a relative scale, e.g. prediction of the number of mRNA molecules in case of the JAK2/STAT5 model is not possible. At least one measurement on an absolute concentration scale is necessary to enable predictions on an absolute scale. When using the reparameterized model, the parameter that corresponds to 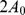 can in this case securely be fixed to an arbitrary value, without restricting the dynamics of the model.

All results shown before were based on reparameterized models with disentangled parameters. In order to evaluate the positive effect of the disentanglement of parameters on the efficiency of parameter estimation, 100 independent runs using the best method were performed without reparameterization (Figure 5c,d). For the JAK2/STAT5 model, the optima are reached more efficiently and reliably by fitting the reparameterized model. Since the Epo receptor model is smaller, the performance by reparameterization has not improved significantly in this case.

### Considering uncertainties in model predictions

As discussed above, it is important that parameter estimation can be performed reliably and efficiently. However, if specific model predictions are required, finding the set of optimal parameter values is only the starting point. The measurement uncertainties of the experimental data have to be propagated to the estimated parameters and, in turn, to the model predictions. In general, there are two approaches to deal with uncertainties:

1. Uncertainties can be investigated in terms of the analysis of *identifiability* of model parameters [26], of *observability* of the predicted dynamics [27] and the calculation of *confidence intervals* [28]. It is of utmost importance that experimental data is sufficiently informative. Otherwise, the problem of parameter *non-identifiability* can arise [26]. Non-identifiability of a parameter indicates that its value cannot be determined given the available data, i.e. confidence intervals are infinite. As a consequence the model dynamics and predictions affected by this parameter may not be determined, i.e. *non-observable* [27], and subsequent analysis might not be reliable. To overcome this problem, an iterative cycle of identifiability analysis, experimental design and performance of the proposed measurements can be applied. For investigation of confidence intervals and identifiability of parameters the profile likelihood approach can be used [26]. For each parameter 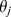 a profile
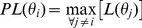
(9)can be calculated individually. The profiles break down the uncertainty contained in the high-dimensional likelihood to a footprint in one dimension. A perfectly flat profile indicates a structural non-identifiable parameter. Using a desired confidence level, practically non-identifiable and identifiable parameters can be distinguished by the profiles. In the latter case, the confidence intervals of the estimate are finite and can be directly obtained from the profile. Since only one-dimensional profiles are calculated, this approach for identifiability analysis is computationally feasible for large models and its results are easy to visualize and to interpret. A similar concept, the prediction profile likelihood, can be used to study observability of model predictions [27].
2. Alternatively, the uncertainties in the parameter estimates and in model predictions can be evaluated by applying *Markov chain Monte Carlo* approaches [29] that facilitate the sampling from the posterior distribution of parameters and of model predictions. While the sampling approach can yield enlightening results it may come at a high computational cost, especially if the parameter space in question is high-dimensional.

Both approaches were recently described and compared in the context of quantitative dynamic models [30]–[32]. The efficiency of the MCMC sampling for the 115 dimensional parameter space of the JAK2/STAT5 model benefits from the efficient parameterization of the model and numerical implementation of the ODE solver. In this setting, reliable results can still be obtained within acceptable computation time. It was demonstrated that they yield similar results, however (1) has superior performance in practice. If the accuracy in the desired predictions is insufficient, *experimental design* can be used to generate additional experimental data that enhances the predictive power of the model. The iterative cycle between model calibration, uncertainty analysis and experimental design was recently demonstrated in detail for the Epo receptor model [25].

## Conclusions

We presented a comprehensive discussion and comparison of methods used for quantitative dynamic modeling, employing two recent examples of relevant size and impact. Implementation of the modeling examples and source code of the methods are freely available and can be used as reference for future applications (Materials and Methods: Software implementation).

For successful model calibration and the estimation of unknown model parameters, it is crucial to have a realistic estimate of the measurement noise of the experimental data. We recommend to estimated the parameters that characterize the measurement noise of the experimental data simultaneously with the parameters that determine the model dynamics. This approach enables determination of the quality of experimental data in an objective and automated manner. Avoiding preprocessing of the experimental data represents substantial progress towards a statistically more reliable procedure, especially in the case of low replicates, which is typical for applications in biology. Furthermore, the assessment of measurement noise in the case of single repetition experiments is feasible. The additional computational cost of that arises from the extra parameters defining the measurement noise parameters is comparatively small.

Numerical optimization used for the estimation of unknown model parameters is a challenging task. We compared the performance of 15 stochastic, deterministic and hybrid optimization algorithms. The results show that multi-start deterministic optimization using the sensitivity equations for the calculation of derivatives significantly outperforms all other tested algorithms. In our evaluation the performance of stochastic optimization algorithms was surprisingly low compared to the hybrid and fully deterministic optimization algorithms. The stochastic optimization algorithms considered here do not make use of derivative information whereas the hybrid and fully deterministic optimization algorithms do. It is documented in [33] that there are considerable disadvantages in not using derivative information. So one cannot expect the performance of derivative-free methods to be comparable to those of derivative-based methods.

Each of the stochastic algorithms has multiple tuning parameters that can improve their performance for a specific application. It is important to note that all algorithms (stochastic, deterministic as well as the hybrid algorithms) were applied with default settings that are recommended for generic applications to avoid manipulation of the results. Based on the example models presented here, our aim was to report the performance of these methods in an unsupervised and hence unbiased way. This reflects the situation in a real application for which no prior knowledge about the optimization problem is available and yields in our opinion the most objective performance evaluation.

Deterministic optimization algorithms make use of derivative information, typically through finite difference approximations. Unfortunately, these approximations tend to yield numerically unstable results for dynamic models [23]. For the two quantitative dynamic modeling examples employed here, we explicitly demonstrated that this leads to unreliable results. Hence, our method of choice for calculating derivatives for dynamic models is the simultaneous solution of the sensitivity equations. We have shown this strategy to be more reliable and efficient.

We report an additional advantage of our performance evaluation of optimization routines by systematic comparison of repeated parameter estimations. It has previously been very difficult to decide whether an unsatisfactory parameter estimation run was due to a local optimum or to a premature termination of the optimizers. Reasons for this premature termination include numerical inaccuracies in the calculation of derivatives. In our definition, true (local) optima are indicated by the fact that several parameter estimation runs result in the same (local) optima in terms of the objective function. Parameter values, however, can be different, if the parameters are non-identifiable. Surprisingly, based on this definition of optima, the number of local optima was rather limited in the examples we have tested so far.

Stochastic optimization algorithms are often applied based on the argument that the objective function contains a plethora of local optima. We showed that deterministic optimization algorithms with inaccurate approximations of derivatives could cause spurious local optima. Instead of employing stochastic optimization algorithms to solve this artificial problem, we suggest to use the sensitivity equations for a reliable calculation of derivatives. True local optima can arise due to insufficient amount and quality of experimental data in combination with the non-linearity of the models. Our proposed performance evaluation of parameter estimation facilitates the detection of these local optima. Concluding, our results show that the best and most accurate method to reveal true local optima and the global optimum is a deterministic derivative-based optimization using the sensitivity equations for calculating derivatives in combination with a multi-start strategy based on Latin hypercube sampling of the initial guesses for the parameters. Furthermore, we show that an alternative parameterization of the dynamic model has superior performance for larger applications.

Finally, we discussed the importance of uncertainty analysis of the estimated model parameters and of the model predictions. For the examples discussed here, challenges in quantitative dynamic modeling are surmounted allowing useful insights into the underlying biology [2], [7].

## Materials and Methods

### Latin hypercube sampling

In order to make deterministic optimization algorithms more robust against local optima a “multi-start” approach can be used. Here, many independent optimization runs from different initial guesses are performed.

**Figure 10:**
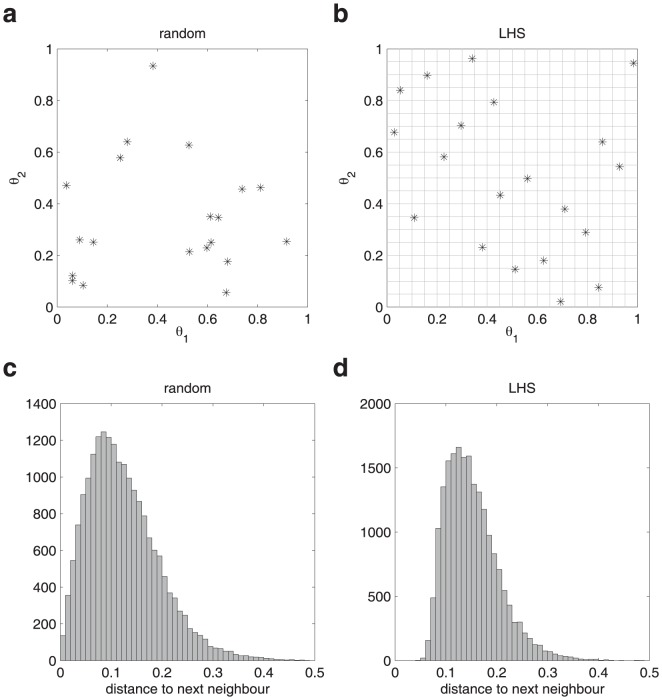
Comparison of random sampling and Latin hypercube sampling (LHS) for the generation of initial parameter guesses. (a) One realization of twenty samples drawn randomly in a two dimensional parameter space is shown. (b) Twenty samples drawn by LHS. (c,d) Euclidean distance to the nearest neighbor parameter values for one thousand repeated generations of twenty samples in a 2D parameter space.

For the generation of the initial parameter guesses purely random sampling or Latin hypercube sampling [17] can be used (Figure 10a,b). Drawing 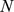 Latin hypercube samples in 2D can be illustrated by dividing the space into 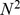 boxes. For the first sample, one box in the first row is selected and then drawn from within this box randomly. For the second sample, one box in the second row is selected, except of the columns that have previously already been drawn from, and then drawn from within this box randomly.

Latin hypercube sampling is favorable compared to purely random generation because Latin hypercube sampling prohibits two randomly selected starting points from being accidentally close to each other (Figure 10c,d). In contrast, for a random generation samples are sometimes very close to each other. Therefore, Latin hypercube sampling provides a better coverage of the space.

### Stochastic optimization algorithms

The performance of twelve different stochastic optimization algorithms [18] were investigated (Table 1). This includes a standard evolutionary optimizer, an evolutionary strategy with covariance matrix adaption, a standard genetic algorithm, particle swarm and adaptive particle swarm optimization, a differential evolution algorithm, a hill climbing strategy, as well as a random Monte-Carlo search. In addition, clustering-based optimization approaches like cluster-based niching evolutionary strategy, clustering hill climbing strategy, an evolutionary strategy with increasing population size, a cluster-based niching genetic algorithm, as well as a population based incremental learning algorithm are available.

**Figure 11:**
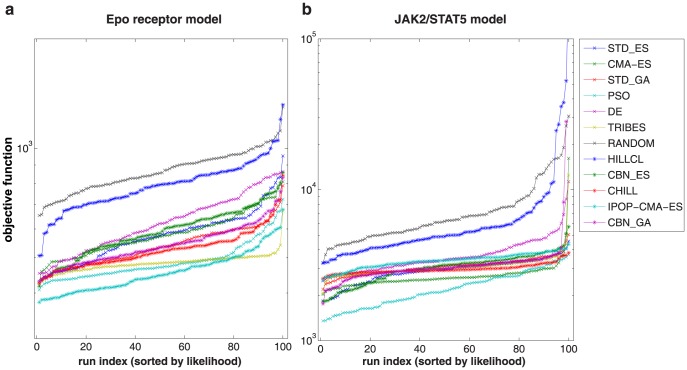
Comparison of optimization performance for stochastic optimization algorithms. The figure shows 100 independent runs by each of the considered algorithms (Table 1) on the x-axis, sorted by the respective value of the objective function. For illustrative reasons, the y-axis was shifted by a constant (Figure 5b).

One hundred independent runs of parameter estimation are performed with each of the twelve algorithms (Figure 11). The results show that the particle swarm optimization algorithm performs best for the Epo receptor model and second best for the JAK2/STAT5 model. The evolutionary algorithm with increasing population size performed best for the Epo receptor model but does not show good performance for the larger JAK2/STAT5 model. Ideally, the algorithms should find reproducibly the global optimum, i.e. return the same value of the objective function multiple times (Figure 5a,b). However, none of the algorithms is able to do so for neither of the examples.

### Software implementation

The model equations and experimental data for the Epo receptor and the JAK2/STAT5 models are available from the original publications [2], [7]. Here, we additionally provide user-ready implementations in a novel computational format for quantitative dynamic modeling that we introduce here. The novel format is compliant with the SBML standard [34], i.e. models can be imported and exported. It is especially tailored to quantitative dynamic modeling applications as are presented here. The software is based on MATLAB (R2011a, The Mathworks Inc., Natick, MA), its source code is freely available from the hosting site Bitbucket: https://bitbucket.org/d2d-development/d2d-software/wiki/Home. It includes a parallelized implementation of the ODE solver CVODES [14] that also allows to solve the sensitivity equations for deterministic optimization and implementation of all modeling concepts and algorithms presented here. The modeling examples [2], [7] are easily accessible by the provided software implementation and allow for testing novel methods and for the characterization of their performance. They can also be used as a reference for further applications.

### Equations for Epo receptor model

The rate equations of the reactions are
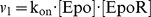


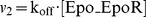


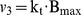


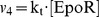


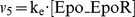


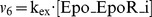


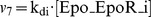


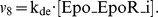



The ODE systems is composed out of the rate equations by
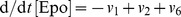


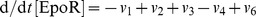


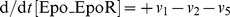


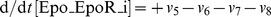


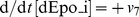


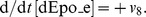



The initial condition are
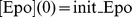


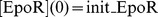


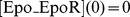


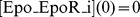


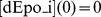


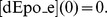



The parameter transformation that decouple the parameter are
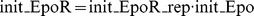


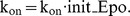



In the re-parametrized formulation, the scale of the dynamics is only determined by the parameter 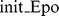 that solely carries the units of concentration.

### Equations for JAK2-STAT5 model

The rate equations of the reactions are
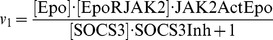


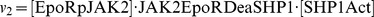


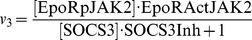


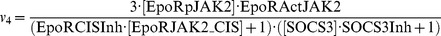


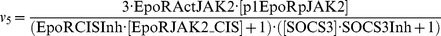


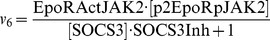


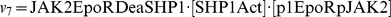


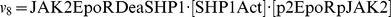


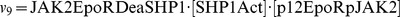


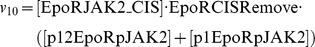





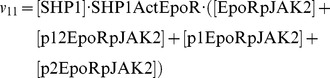


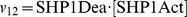







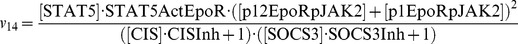


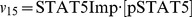


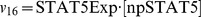


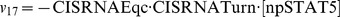


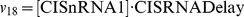


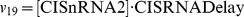


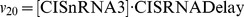


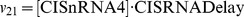


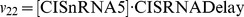


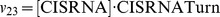


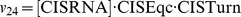


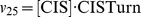


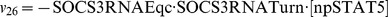


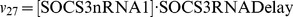


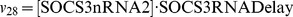


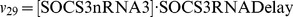


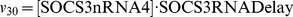


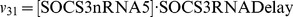


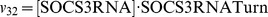


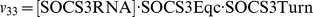


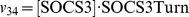



Reactions 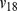 to 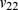 and 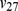 to 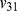 account for a delay that summarize the processing steps of the mRNA by a linear chain of reactions [35] with common rate constant CISRNADelay and SOCS3RNADelay, respectively. The ODE systems is composed out of the rate equations by
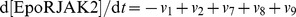


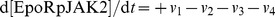


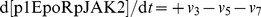


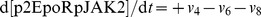


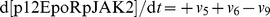


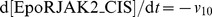


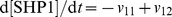


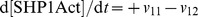


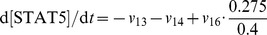


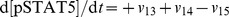


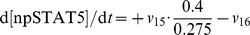


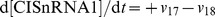


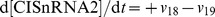


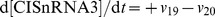


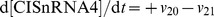


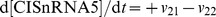


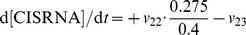


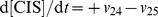


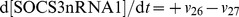


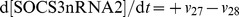


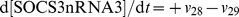


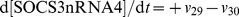


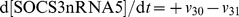


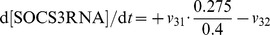


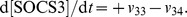



The volume factors 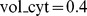 pl and 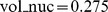 pl account for transitions between different compartments and are determined experimentally. The species npSTAT5, CISnRNA1–5 and SOCS3nRNA1–5 are located in the nuclear compartment, the remaining species in the cytoplasmatic compartment. The initial condition are set to zero except for
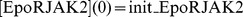


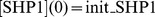


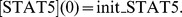



The parameter transformation that decouple the parameter are
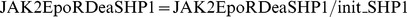


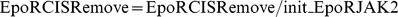


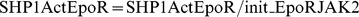


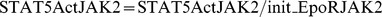


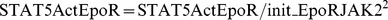


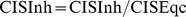


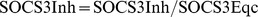


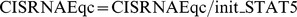


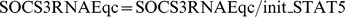


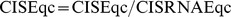


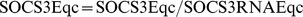



In the re-parametrized formulation, the scale of the dynamics is only determined by the parameters 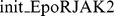, 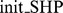, 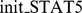, 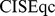 and 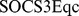 that solely carry the units of concentration.
