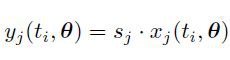# Correction: Lessons Learned from Quantitative Dynamical Modeling in Systems Biology

**DOI:** 10.1371/annotation/ea0193d8-1f7f-492a-b0b7-d877629fdcee

**Published:** 2013-12-09

**Authors:** Andreas Raue, Marcel Schilling, Julie Bachmann, Andrew Matteson, Max Schelker, Daniel Kaschek, Sabine Hug, Clemens Kreutz, Brian D. Harms, Fabian J. Theis, Ursula Klingmüller, Jens Timmer

The fifth author's name was spelled incorrectly. The correct name is: Max Schelker. The corrected citation is:

Raue A, Schilling M, Bachmann J, Matteson A, Schelker M, et al. (2013) Lessons Learned from Quantitative Dynamical Modeling in Systems Biology. PLoS ONE 8(9): e74335. doi:10.1371/journal.pone.0074335

In equation 7, many additional factors of 2 were added incorrectly. Please see the correct equation 7 here: 


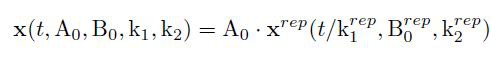


In equation 8, '2A_0' should only read 'A_0.' Please see the correct equation 8 here: